# A Tactile Virtual Reality for the Study of Active Somatosensation

**DOI:** 10.3389/fnint.2020.00005

**Published:** 2020-02-18

**Authors:** Arindam Bhattacharjee, Diljit Singh Kajal, Alessandra Patrono, Yiwen Li Hegner, Massimiliano Zampini, Cornelius Schwarz, Christoph Braun

**Affiliations:** ^1^Werner Reichardt Center for Integrative Neuroscience, University of Tübingen, Tübingen, Germany; ^2^Hertie Institute for Clinical Brain Research, Department of Cognitive Neurology, University of Tübingen, Tübingen, Germany; ^3^MEG Center, University of Tübingen, Tübingen, Germany; ^4^DiPSCo, Department of Psychology and Cognitive Sciences, University of Trento, Rovereto, Italy; ^5^Hertie Institute for Clinical Brain Research, Department of Neurology and Epileptology, University of Tübingen, Tübingen, Germany; ^6^CIMeC, Center for Mind/Brain Sciences, University of Trento, Rovereto, Italy

**Keywords:** active touch, passive touch, active perception, somatosensory, virtual reality, Piezo-electric stimulation, active sensing, threshold

## Abstract

Natural exploration of textures involves active sensing, i.e., voluntary movements of tactile sensors (e.g., human fingertips or rodent whiskers) across a target surface. Somatosensory input during moving tactile sensors varies according to both the movement and the surface texture. Combining motor and sensory information, the brain is capable of extracting textural features of the explored surface. Despite the ecological relevance of active sensing, psychophysical studies on active touch are largely missing. One reason for the lack of informative studies investigating active touch is the considerable challenge of assembling an appropriate experimental setup. A possible solution might be in the realm of virtual tactile reality that provides tactile finger stimulation depending on the position of the hand and the simulated texture of a target surface. In addition to rigorous behavioral studies, the investigation of the neuronal mechanisms of active tactile sensing in humans is highly warranted, requiring neurophysiological experiments using electroencephalography (EEG), magnetoencephalography (MEG) and/or functional magnetic resonance imaging (fMRI). However, current neuroimaging techniques impose specific requirements on the tactile stimulus delivery equipment in terms of compatibility with the neurophysiological methods being used. Here, we present a user-friendly, MEG compatible, tactile virtual reality simulator. The simulator consists of a piezo-electric tactile stimulator capable of independently protruding 16 plastic pistons of 1 mm diameter arranged in a 4 × 4 matrix. The stimulator delivers a spatial pattern of tactile stimuli to the tip of a finger depending on the position of the finger moving across a 2-dimensional plane. In order to demonstrate the functionality of the tactile virtual reality, we determined participants’ detection thresholds in active and passive touch conditions. Thresholds in both conditions were higher than reported in the literature. It could well be that the processing of the piston-related stimulation was masked by the sensory input generated by placing the finger on the scanning probe. More so, the thresholds for both the active and passive tasks did not differ significantly. In further studies, the noise introduced by the stimulator in neuromagnetic recordings was quantified and somatosensory evoked fields for active and passive touch were recorded. Due to the compatibility of the stimulator with neuroimaging techniques such as MEG, and based on the feasibility to record somatosensory-related neuromagnetic brain activity the apparatus has immense potential for the exploration of the neural underpinnings of active tactile perception.

## Introduction

Tactile texture perception is typically an active process; i.e., animals voluntarily move specific body parts (e.g., fingertip in humans or whiskers in rodents) across a surface to generate dynamically changing tactile stimuli. In other words, active tactile sensing is a confluence of two processes: (a) precisely controlled movements of the sensory surface; and (b) the perception of the emergent tactile stimuli. Despite the ecological relevance of the active component of tactile sensing, in research and in clinical tests predominantly passive sensing is assessed during which tactile stimuli are delivered to a stationary finger. Whereas passive touch experiments are relatively easier to implement, and high-precision controlled stimulation is possible, recent studies in humans suggest an intricate link between motor processing and the somatosensory system (Limanowski et al., 2019). For example, Simões-Franklin et al. (2011) showed in an functional magnetic resonance imaging (fMRI) study investigating the exploration of sandpaper textures that the active compared to passive condition elicited stronger activations of the primary somatosensory region. Therefore, passive touch studies alone might fail to fully capture the role of the somatosensory system. One of the reasons for fewer studies in active tactile sensation could be the technical challenge of delivering well-controlled stimuli while the participant’s finger is moving. Previous commendable approaches to study active tactile sensation used equipment that was mostly restricted to those studies alone (Phillips et al., 1990; Vega-Bermudez et al., 1991; Cascio and Sathian, 2001; Gamzu and Ahissar, 2001). Thus, there is a need for a versatile tool that could create a virtual tactile reality, which will eventually provide the opportunity to present a variety of texture stimuli to the participant.

Whereas there are some human behavioral studies on active somatosensation, there are even fewer studies investigating the neuronal mechanisms of active tactile perception in humans. Investigations using neuroimaging tools [e.g., by using magnetoencephalography (MEG) or fMRI] require equipment that is compatible with the testing environment, and does not generate electrical noise that might corrupt or interfere with the recorded neurophysiological signals. In other words, there is a need for a tool that goes beyond psychophysical experiments and overcomes the limitations of compatibility in neurophysiological and neuroimaging experiments. To that end, we developed a tactile virtual reality setup compatible with MEG and that could be used for psychophysical experiments as well.

Typically, the manual presentation of different textures by the experimenter is slow and the possibility of unintended experimenter bias is high. Therefore, an automated and bias-free presentation of stimulus surfaces for efficient estimation of vibrotactile thresholds is always preferable. The novel tactile virtual reality setup presented here motivates two primary goals—(a) naturalistic finger sensing on textured surfaces (ecological validity); and (b) computer-controlled equipment to present the textured target stimuli efficiently (without the requirement of manually exchanging different surface probes).

**Figure 1:**
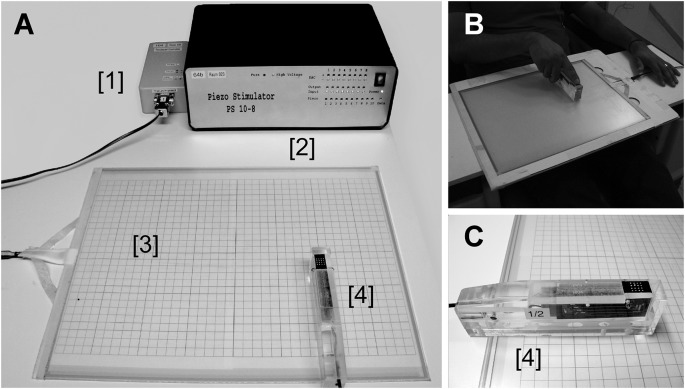
Panel **(A)** shows four components of the setup; the touchpad control unit [1], the piezo control unit [2], the touchpad [3], and the scanning probe housing the piezoelectrically driven pistons. Panel **(B)** depicts the tactile virtual reality in operation. Panel **(C)** shows a closer picture of the scanning probe . Note, to increase the visibility of the transparent touchpad in this figure it was placed on a sheet of paper with printed gridlines. During experiments with participants, the sheet was removed.

When we move our finger on textured surfaces, the interaction between the fingertip and the surface elicits vibrotactile stimulation (Hollins et al., 2002; Bensmaia and Hollins, 2003), which allows us to infer the underlying texture. Here, we worked under the assumption that it should be possible to recreate the textures virtually by presenting the corresponding vibrotactile stimulation pattern to the fingertips if different textures generate different kinds of vibrotactile sensory input (Bensmaia and Hollins, 2003; Manfredi et al., 2014). Our tactile virtual reality setup presented here consists of three main components—a resistive touchpad, a scanning probe (which the participant holds), and the control system. The scanning probe contains a 4 × 4 matrix of piezo-electrically driven pistons covering a square skin area of 1 × 1 cm^2^ (Figure 1A). To explore a virtual surface, a user moves the scanning probe over the touchpad with the probing fingertip placed over the piston matrix while the remaining fingers hold the body of the scanning probe. Depending on the position of the scanning probe on the touchpad, pistons are activated to simulate a predefined surface (i.e., the stimulus delivery depends on the user’s choice of placing the scanning probe on the touchpad). The piston matrix mimics locally the vibrotactile stimulation that is perceived while touching the surface of real objects. Moving the scanning probe thus alters the tactile stimulation pattern. While fast movements of the probe translate into higher stimulation frequencies, slow movements result in lower frequencies.

We consider this study as an introduction of a new method that is suitable for investigating active tactile perception in humans. Therefore, we first describe the technical details of the virtual tactile reality, next we demonstrate its functionality and applicability by implementing one behavioral and three MEG experiments. In Experiment 1, we estimated and compared psychophysical detection thresholds in the active (hand movement) and passive (no movement) conditions. In Experiment 2, we conducted a series of noise measurements to test the compatibility of the touchpad and the piezo-electric scanning probe in the MEG environment. In Experiment 3, we compared the evoked responses elicited by the stimulation of the scanning probe at the fingertip when the participant actively moved the scanning probe across the touchpad against the same stimulation but without the hand movement. In Experiment 4, neuromagnetic steady-state responses were recorded while actively scanning the texture of a grating-like surface. We emphasize here that the purpose of the current study is to demonstrate the effectiveness of the tactile virtual reality setup rather than to investigate active tactile perception in humans, *per se*, which is a topic for future experiments.

### Method: Virtual Tactile Reality

The custom-built (MEG Center, University of Tübingen, Germany) virtual tactile reality consists of five parts (Figures 1A,C): stimulation computer with Linux Operating System, a standard 400 × 325 mm 4-wire resistive touchpad (KEYTEC KTT-191LAM, Garland, TX, USA), a touchpad control unit (in-house built), a piezo-electric control unit (in-house built), and a scanning probe. The scanning probe consists of two sets of commercially available piezo-electric Braille elements (Metec AG, Stuttgart, Germany); each set has a 4 × 2-piston matrix.

**Figure 2:**
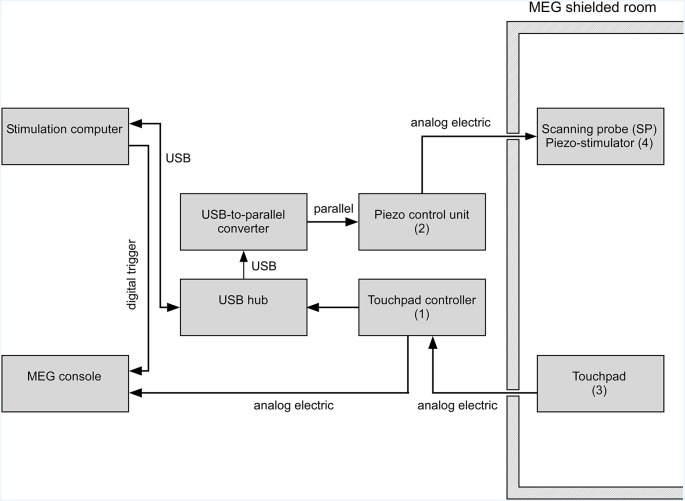
Schematics of the virtual tactile stimulator operated in a magnetoencephalography (MEG) environment. While touchpad (Figure 1A item 3) and scanning probe (Figure 1C item 4) are inside the shielded magnetic room, all other parts are kept outside. The position of the scanning probe on the touchpad is determined by the touchpad controller (Figure 1A item 1) and sent to the stimulation computer *via* a USB connection. Depending on the scanning probe position the somatosensory stimulation pattern is sent to the piezo control unit (Figure 1A item 2). The control unit generates the supply voltage individually for each piezo element. Individual piezo elements can move the pistons of the 4 × 4 stimulation matrix individually in a graded fashion. The position of the scanning probe on the touchpad is recorded continuously together with stimulus information by the MEG system.

The position of the scanning probe on the touchpad determines the spatial pattern of the tactile stimulation to be delivered to the fingertip (see Figure 2 for command flow). By using a standard four wire resistive touchpad as a virtual surface, we identify the position of the scanning probe. The touchpad uses an input voltage of 50 mV instead of the standard supply voltage of 5 V; this decrease in voltage reduces the current in the touchpad which in turn decreases MEG artifacts and prevents malfunctioning of the MEG sensors. To further minimize the artifacts generated by the touchpad, we use a driver chip (74HC244) with a separate voltage filter. Galvanic decoupling provided electrical safety and reduced power line artifacts. Additionally, there is also a second-order low pass filter between the electronics of the touchpad and the control unit. The touchpad connects to the touchpad control unit (see Figure 2) through twisted low magnetic noise wires that minimize distortions of MEG signals during neuromagnetic recordings. The controller unit operates at exactly 600 Hz resulting in an update of the piston protrusion every 1.6 ms. The 600 Hz frequency allows MEG artifacts only to occur at that frequency and its harmonics. The touchpad and the piezo-electric control unit connected to the stimulation computer *via* standard “high speed” serial USB ports that guarantee reduced response latencies.

The stimulation patch (Figure 1C, black surface with holes containing white piston arrays) in the scanning probe, which covers a skin area of 10 × 10 mm^2^, consists of a set of two refreshable piezo-electric 4 × 2 Braille elements, thus creating a 4 × 4 configuration of 16 pistons. The distance between the adjacent pistons is 2.5 mm. Individually each piston protrudes by graded activation of the piezoelectric crystals in the range of 0–1 mm with a resolution of 0.01 mm. The piezoelectric elements are operated at a sampling rate of 1.2 kHz. The tips of the non-activated pistons remain flush with the top surface of the scanning probe. The protrusion of the individual piston and a group of pistons is controlled by the stimulation computer connected to the piezo-control unit.

The sequence of steps that leads to tactile stimulation after a user puts down the scanning probe on the touchpad is as follows: the touchpad captures the scanning probe position and transmits the coordinates to the stimulation computer *via* the touchpad controller unit as a digitally encoded “finger” position signal (Figure 2). The touchpad controller also sends an analog electrical signal which is recorded by the data acquisition system (MEG system). The touchpad controller separates the electric potentials between the touchpad and the connected recording device (MEG system; Figure 2). The galvanic decoupling ensures electrical safety and reduces power line artifacts. The USB-hub is a standard device and helps to reduce the latencies introduced by the setup. The in-house built USB-to-parallel converter provides a versatile interface between the stimulus control computer and the piezo-control unit. The piezo-control unit controls the protrusion or elevation of the pistons of the piezo-stimulator. It receives its input *via* a parallel port (Figure 2). The stimulation computer controls the sequence of stimulation and sends triggers to the MEG console to synchronize the stimulation with any neuroimaging recordings. The stimulation computer also receives and stores the information of the position of the scanning probe on the touchpad in a log file, updates the position-dependent tactile pattern of the piezo-stimulator *via* the piezo-control unit and controls the overall experimental flow. For further technical information, please contact our laboratory.

Below, we present four experiments as applications that demonstrate the functionality of the virtual tactile reality equipment described above. Within each experiment, we present the rationale, the experimental procedure, obtained results, and a brief discussion.

## Experiment 1: Active and Passive Somatosensation

Whereas movement is critical for texture perception, an obvious question in active perception is: what is the perceptual fate of a tactile stimulus without the movement? Does the tactile stimulus that is generated from participants’ active movement (*active condition*) perceptually feel the same when that stimulus is presented passively to them? Evidence for altered processing of somatosensory stimuli during active movement in contrast to *passive perception* comes from studies that reported an elevation of detection thresholds during active perception (Chapman et al., 1987) of tactile stimuli—a phenomenon known as “sensorimotor gating” (Braff and Geyer, 1990; Cromwell et al., 2008). Note that, active movement introduced in these studies was more akin to ballistic movements rather than active sensory exploration. Nevertheless, the critical issue here is the movement of the sensor during the process of stimulus acquisition by the nervous system.

To investigate gating effects, we asked participants to explore the patterns of virtual vertical ridge textures in an active condition by moving the scanning probe across the touchpad in front of them; depending on the scanning probe location (i.e., the finger location) we delivered corresponding task-dependent tactile stimulus to their index finger. We asked the participants to find the location of the just noticeable stimulus by moving the scanning probe across the touchpad. Threshold intensities obtained in the active condition were compared against that in the passive condition; during the passive condition the hand was stationary and the piston protrusion (elicited by the participant’s scanning behavior) recorded in the active condition was replayed. Participants reported the perception of stimuli by key presses.

### Method (Experiment 1)

#### Participants

Sixteen participants participated in the study (14 males and two females, age M ± SD: 24.31 ± 3.83). All participants, except one, were right-handed—determined by the Edinburgh Handedness Inventory (Oldfield, 1971). All participants were sensory and neurologically healthy (self-reported) and were not under any medication. All participants volunteered in the study and gave written informed consent in accordance with the Declaration of Helsinki 1964 (most recently amended in 2013, Fortaleza). The protocol of this study was approved by the Ethics Committee of the Medical Faculty of the University of Tübingen and the study was carried out in accordance with their recommendations.

#### Stimulation

**Figure 3:**
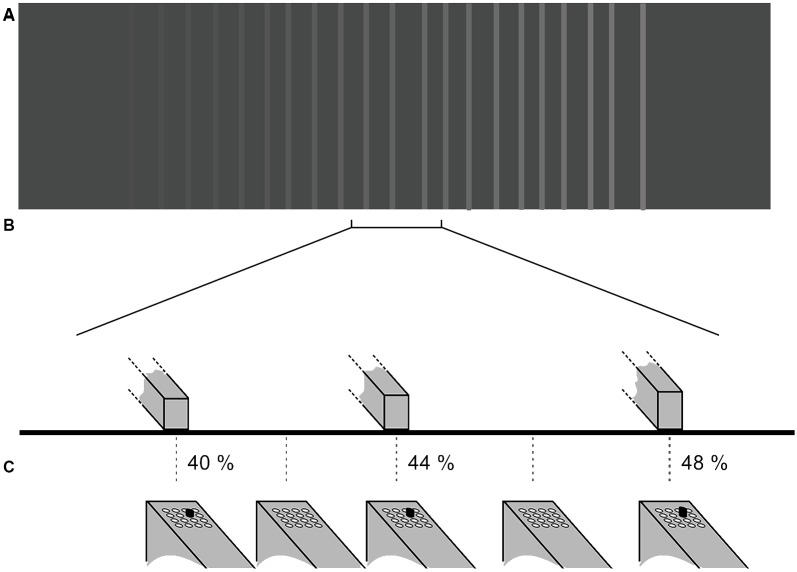
Tactile stimulation is provided to the fingertip of the subject based on the texture image presented on the virtual reality pad. Plots show (not to scale) an example of **(A)** the pattern of the gratings on the virtual pad (not visible to the participant) and **(B)** the height of the three ridges within the bracketed section. The gradient of stripe intensities represents the elevation of the virtual lines. In **(C)** the tactile stimulation pattern presented to the fingertip *via* the hand-held tactile stimulator is shown (4 × 4 piston matrix). In this experiment, only the same single-piston marked in black moved up or down depending on the scanning probe position on the touchpad. The protrusion of the piston is given in percent.

Prior to the beginning of the experiment, using Tcl/TK we generated gray level images, which were translated into grating-like virtual textured surfaces. Depending on the position of the scanning probe on the touchpad (and thus the corresponding virtual surface sector), the participants received the corresponding tactile stimulus on their index finger of their dominant hand. The virtual textures served as templates for the stimulation (see Figure 3) such that the lighter the shade of gray the higher was the protrusion of the piston—white stripes represented the positions on the touchpad where the pistons were 100% protruded. Intermediate protrusions of the piston were coded by corresponding gray-levels. The size of one pixel on the image is equivalent to 0.25 mm distance on the touchpad; the total displayed image contains 1,500 × 1,100 pixels or 375 mm × 275 mm. The extension of the touchpad was large enough in order not to restrict the participants’ scanning probe movements to a small section. The virtual surface consisted of a texture of irregularly spaced vertical ridges that were elevated with respect to the surface of the cast of the scanning probe (see Figure 3). The elevation of the ridges changed steadily with space in the horizontal (left-right) direction. The distance between stripes varied randomly from between 7.5 mm and 12.5 mm. The width of the line was 2.5 mm. Each trial used a distinct image file (Figure 3A). A new set of images was created for each participant at the beginning of each experiment.

#### Experimental Procedure

**Figure 4:**
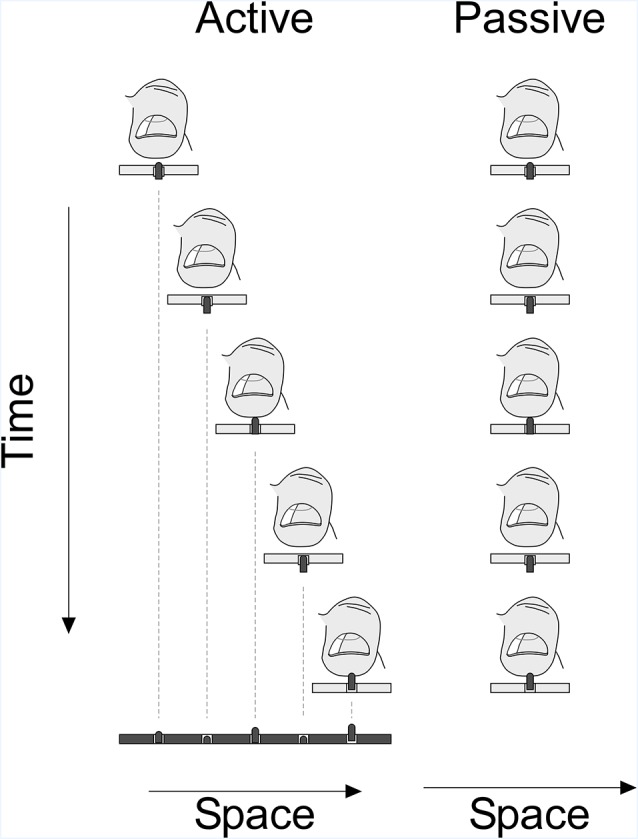
Experimental paradigm: plot showing the active and passive condition of the experiment. In the active phase, participants scanned the virtual space using the stimulation probe and were stimulated depending on the position of the probe and the virtual surface. In the passive phase, the scanning probe stimulated the virtual space at the index finger with a stimulation sequence identical to the sequence in the active condition.

Participants sat upright on a chair facing the touchpad in a well-lit room. They held the scanning probe in their dominant hand such that the glabrous part of the distal phalanx of their index finger covered the tactile stimulation patch (Figure 1B). Each participant was tested in two different sessions on different days—day 1, active and day 2, passive condition, respectively. Because the stimuli of the passive condition (Figure 4) were a copy of the stimulation sequence that participants had experienced in the active condition, the active condition had to precede the passive condition.

In the active condition, participants moved the scanning probe horizontally across the touchpad; the position of the scanning probe on the touchpad was recorded for online stimulus presentation and offline data analysis. In each trial, a different virtual texture pattern with random inter-ridge distances (between 7.5 mm and 12.5 mm) was chosen. Moreover, the gradient of amplitude change (high to low amplitudes or vice versa) from the left to the right side of the touchpad was randomized across trials. The trial by trial randomization of the gradient of change and the spacing of stripes ensured that the participants could not memorize a location or use a predetermined position on the touchpad as a response.

Each trial started when the participant brought the scanning probe to the “home position” at the left margin of the touchpad. Participants initiated the trial by pressing the “Enter” button on the computer keyboard with their non-dominant hand. This keypress also started the presentation of auditory white noise delivered *via* head-phones to the participants for the whole duration of the trial to mask any auditory cues generated by the piezoelectric stimulator. During any trial, if the touchpad lost contact with the scanning probe, a series of low-frequency tones alerted the participants. A series of high-frequency tones were presented when the touchpad detected more than one contact (i.e., a contact other than that of the scanning probe). In both cases, the tones continued until the participant rectified the situation. Participants were instructed to move the scanning probe across the virtual surface from left to right and back in a self-paced manner to find the location at which they felt that the tactile stimulus was just detectable. Participants reported their response, i.e., perception of the just noticeable elevation of the surface stripes, by pressing the “space-bar” on the keyboard of the stimulation control computer with the non-dominant hand. Participants were asked to double-check before reporting their perception, by scanning the underlying virtual surface back and forth, and respond only when they were sure. From the position of the scanning probe, we inferred the corresponding stimulus intensity that we later used to determine their threshold. Pressing the “space-bar” marked the end of the trial, which also turned off the white noise. Note that during a single trial a considerable number of stimuli (~50–100) with different amplitudes were applied depending on how the scanning probe was moved. Depending on the movement speed the stimulation frequency varied between ~2 Hz and 20 Hz. Scanning probe position, stimulation amplitude and status of the space-bar were recorded at a sampling rate of 100 Hz. After responding the participants returned to the “home position.” Participants were encouraged to take short breaks whenever they needed during inter-trial intervals. We recorded four blocks of 20 trials each, resulting in a total of 80 trials for each participant. Each block lasted for approximately 15 min including rest periods.

In the passive condition (implemented on another day), participants’ tactile detection threshold was determined using a more standardized approach during which the finger rested on the stimulation area of the scanning probe which was placed at the home position on the left side of the touchpad; the hand remained stationary during the trial. The tactile stimulation for active and passive conditions was delivered to the same fingertip. We replayed exactly the temporal pattern of stimulation that was recorded during the active touch condition. In other words, when participants moved their hand holding the scanning probe back and forth in the active condition the stimulus intensity decreased and increased accordingly. In the passive condition, the same sequence of intensities with the same timing as in the active condition was replayed. During the passive condition, the participants reported the perception of stimuli by pressing the space-bar on the keyboard of the computer controlling the experiment. The status of the space-bar signal was registered together with the position of the scanning probe and the stimulation pattern. Since stimuli were presented in a rapid sequence like in the active condition (~2–20 Hz) the spacebar was kept pressed within a trial as long as the stimuli were above threshold. To indicate that they did not feel any stimulation, participants released the space-bar immediately. The stimulation ended when the whole stimulation sequence recorded during the active sensing condition had been replayed. Like in the active condition, we presented auditory white noise during the entire trial. Similarly, participants were free to take a short break after each block. The number of trials and blocks in the passive condition was identical to that of the active condition.

#### Analyses

In order to estimate the sensory detection threshold in the active touch condition, we concatenated all four blocks. In each trial, the stimulation intensity of the last virtual ridge that was crossed by the participant before indicating that the just noticeable height or amplitude had been reached, was taken as the threshold value in that trial. Finally, for each subject, we computed the mean threshold by averaging the threshold values across trials. We estimated the scanning speed by calculating the first derivative of the position of the scanning probe on the touchpad in time.

To estimate the threshold in the passive touch condition, we analyzed the recordings of the time course of the piston protrusion and the status of the space-bar that was continuously recorded at a sampling rate of 100 Hz. Note that the stimulation rate ranged between 2 and 20 Hz. First, we counted in the recordings the number of time points during which the piston was activated, separately for each of the presented piston protrusions. Next, we computed again separately for each piston protrusion the percentage of these time points during which participants also kept the space-bar pressed (i.e., to indicate the perception of the stimulus). Finally, in order to obtain a psychometric function, we fitted a cumulative normal distribution to the percentage of perceived samples expressed as a function of the piston protrusion. We considered the piston protrusion which was perceived during 50% of its presentation (estimated from the fitted cumulative normal distribution) as the participant’s threshold.

Thresholds for active and passive sensation were compared by a *t*-test for dependent variables (using SPSS version 25.0, IBM Corporation, Armonk, NY, USA).

### Results (Experiment 1)

**Figure 5:**
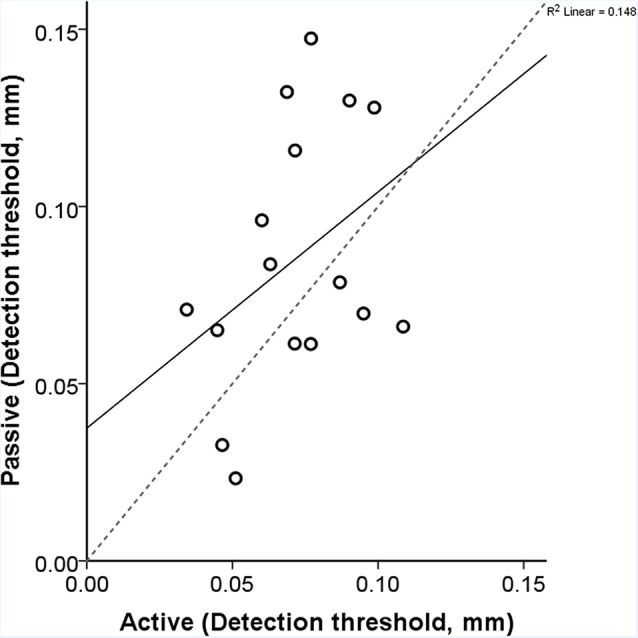
Each participant’s active and passive thresholds are shown here. The gray dashed line is the identity line where the active and passive detection thresholds would be identical. The correlation between the active and passive threshold was 0.385 (*p* = 0.141). The equation of the regression line is y = 0.67x + 0.04.

The obtained thresholds for the active and passive conditions were 0.07 mm (*SD*: ±0.02) and 0.08 mm (*SD*: ±0.04), respectively. We performed a paired-samples *t*-test between the two conditions and found no statistical difference (*t* = 1.581, *df* = 15, *p* = 0.135; Figure 5). During the active condition, we observed that the average maximum speed of exploration/scanning was 87.44 mm/s (*SD*: 45.4 mm/s). The correlation between the speed and active threshold was −0.432 (*p* = 0.095).

### Discussion (Experiment 1)

Through this experiment, we demonstrated that using the equipment presented in this study there exists the possibility to capture movement induced tactile stimuli in an active condition and identically replicate it in a passive condition. Here, we estimated vibrotactile detection thresholds while participants actively scanned a virtual textured surface using their index finger; we then compared these threshold estimates with thresholds obtained in the passive condition when keeping the finger stationary. We found that detection thresholds were consistent for both, the “active” and “passive” threshold estimation procedure. Although results were rather stable across conditions, we obtained relatively higher threshold values compared to those reported in the literature (Gescheider et al., 2009; Gandhi et al., 2011).

How do our thresholds compare against previously reported thresholds? In the glabrous skin, four different types of receptors and corresponding mechanoreceptive afferents encode for every tactile stimulus of which two types are responsible for encoding vibrotactile stimuli—the Meissner corpuscles (connected to the Rapidly Adapting Type 1 afferent) encode low-frequency vibrations in the range of 5–50 Hz, and the Pacinian corpuscles (connected to the Rapidly Adapting Type 2 afferents) encode high-frequency vibrations that range beyond 50 Hz (Bolanowski et al., 1988; Gescheider et al., 2009). The perceived stimulus frequency in the current experiment depended on the participants’ finger scanning speed: the faster they moved, the higher the perceived frequency; the average movement or scanning speed observed in the current study was 87.4 mm/s and the distance between the virtual strips ranged between 7.5 mm and 12.5 mm, which implies that the pulsatile frequency was approximately 12 Hz and 7 Hz respectively; this frequency activates the Meissner corpuscles. However, the waveform frequency of each pulse was around 150 Hz, which typically activates the Pacinian receptor class. Nevertheless, estimated detection thresholds in the current study (approximately 70 μm) are considerably higher compared to the thresholds reported for the Pacinian receptor system, which are typically around 1 μm or less (Gescheider et al., 2009; Yildiz and Güçlü, 2013; Yıldız et al., 2015).

Why do we see thresholds higher than those reported previously? In most vibrotactile studies including the ones mentioned above, the probe providing the stimulus is the only surface that contacted the stimulation area of the skin, or the probe was separated from the adjoining contact surfaces by an annular ring-like gap. In the current scenario, only one piston tip indented the skin surface, whereas the other 15 pistons of the scanning probe were in contact with the skin which could have created a noisy background. Note that the remaining piston tips were flush with the surface and were stationary, i.e., did not vibrate during the trials. Presumably, given the noisy background, detection of the increase in height of the target piston might have been difficult which resulted in elevated thresholds. Another possible reason for the discrepancy in thresholds between the current study and previous studies could be the area of stimulation. In the current study, the area of the stimulus probe that touched the skin was 0.79 mm^2^ which might only activate the Meissner corpuscle system and less optimally the Pacinian corpuscles as has been demonstrated in a psychophysical study by Verrillo (1963). Yet, the threshold reported for the Meissner system is typically below 20 μm (Gescheider et al., 2009). To study how the stimulation should be adjusted and which stimuli should be applied in order to target different receptor types is currently not clear and needs further investigation. Slowly adapting Merkel cells might be best stimulated by broader strips of protruded stimulus pistons inducing lower stimulation frequency than more narrow ridges (Bolanowski et al., 1988). Increasing the number of simultaneously activated pistons will increase the total stimulation area and thus might activate Pacinian corpuscles, which in turn could increase the sensitivity to this stimulation condition.

Is there sensorimotor gating, i.e., an increase in tactile detection thresholds due to the active movement (Chapman et al., 1987; Chapman, 1994)? In the current experiment, we did not find any significant effect of the scanning speed on the detection thresholds; the exploratory speed of the hand during active exploration was on average 87 mm/s. It appears that the detrimental effect of movements on the detection threshold is dependent on the speed of the sensing hand (Cybulska-Klosowicz et al., 2011; Yıldız et al., 2015) and that the speed observed in the current study was much lower than the critical speed (approximately 200 mm/s) at which tactile sensitivity decreases. Moreover, fine texture perception requires movement and sensory gating would impede the whole process of perception.

Finding no threshold difference between active and passive conditions could due to several reasons. It could be argued that the active condition in our setup lacks the interaction between the fingerprint and the tactile surface. Since the finger is placed on the scanning probe the stimulation pattern elicited in the active condition does not differ from the passive condition. The only difference between conditions is the interaction between the sensory input with and without the self-generated hand movement. In other words, in our study, the receptors in the fingertip evoke the same responses in both active and passive conditions, which in turn result in the same percepts and consequently similar thresholds. Furthermore, the lack of difference could be due to different biases introduced in both conditions. In the active condition participants were encouraged to verify the position by revisiting the positions in the surrounding of the presumed threshold location on the touchpad. Since in the passive condition there were no instructions to verify the perceptual decision, we cannot exclude the possibility that the participants developed a bias towards a less conservative threshold identification during the passive sensing task as compared to the active task. The bias finally might have led eventually to higher threshold values for the active condition.

## Experiment 2: Electric Noise in Meg

To test the compatibility of the new device with neuroimaging methods, we carried out noise measurements in our MEG laboratory with the virtual reality apparatus switched both, on and off.

### Method (Experiment 2)

We carried out noise measurements using a whole head system (CTF OMEGA, Port Coquitlam, Canada). Magnetic fields were recorded using 275 first-order hardware gradiometer with a baseline of 5 cm placed around the curvature of the MEG helmet and a set of 9 magnetometers and 18 1st order gradiometers served as reference channels for noise cancelation. Effects of 3rd order software gradiometers and power line notch-filters are also reported. Noise measurements were done without recording a participant’s brain activity. The sampling rate of the recording was 11.719 kHz, and the anti-aliasing lowpass filter was set at 2.93 kHz. While the scanning probe with the in-built piezo stimulator and the touchpad were inside the shielded room (AK3b, Vakuum Schmelze, Hanau, Germany), the other parts of the apparatus were placed outside, about 5 m away from the MEG sensors. We tested whether changes of pressure of the scanning probe onto the touchpad creates artifacts in the MEG recording. To this end, in the switched-on condition, a pneumatically membrane was periodically pressing on the touchpad with a frequency of 1 Hz introducing a well-defined extra touchpad current flow on the touchpad. The position of the membrane was chosen such that the pistons of the piezo-stimulators inserted in the scanning probe, The noise level in the switched-on condition was compared to that in the empty shielded room when the stimulator was switched off and the scanning probe and the touchpad were relocated to outside the shielded room. In total 100 s of data were acquired, which were segmented in epochs of 2 s. For each channel, the offset of the magnetic activity was removed by subtracting the mean activity across the whole epoch. Power spectra for both, the switched-on and the empty room conditions were calculated using a multi-taper Fourier analysis based on discrete prolate spheroidal sequences (Slepian sequences) as tapers implemented in field trip (Oostenveld et al., 2011). In order to study whether artifacts could be reduced in the offline analysis, 3rd order software gradiometers were applied.

### Results (Experiment 2)

**Figure 6:**
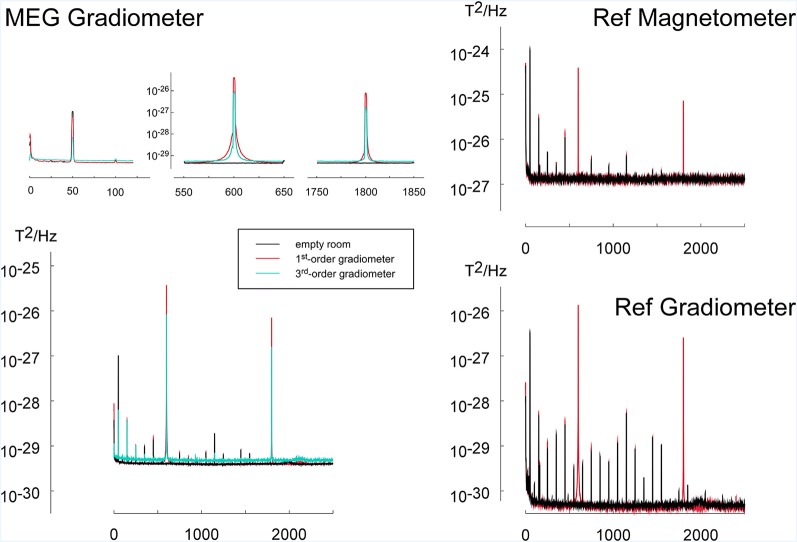
To investigate the noise introduced by the tactile virtual reality device, power spectra during operation of the device (red) were compared to the spectra of the empty room (black). The application of 3rd order software gradiometers (cyan) reduced the power in frequency bands related to the operation of the virtual reality device (600 Hz and harmonics). To mimic the movement of the device by the participant during operation, the scanning probe was pushed onto and released from the touchpad *via* a pneumatic mechanism with a frequency of 1 Hz. The power spectrum during the simulated operation was compared to the noise level of the empty measurement chamber. The MEG measuring the noise spectra consists of three types of magnetic field sensors: (1) MEG first-order axial gradiometers having a baseline of 5 cm are placed in the helmet of the MEG-device and are designed to register participants brain activity. (2) Reference gradiometers (REF Gradiometer) which have a baseline of 2 cm and are placed about 5 cm above the helmet, are used for sensing distant sources of noise. (3) Similarly, reference magnetometers (Ref Magnetometer) located next to the reference gradiometer are also used for noise cancellation. Since reference gradiometers and reference magnetometers are further away from the subject’s brain, they record predominantly environmental noise activity.

During the operation of the virtual tactile reality, noise peaks could be observed at the operation frequency of the touchpad at 600 Hz and its multiples (Figure 6). Furthermore, an increase in activity was visible at 1 Hz, i.e., the frequency at which the pressure on the touchpad was varied locally. Using 3rd order software gradiometers the peaks at these frequencies were reduced yet at the expense of a generally higher noise floor (Figure 6). A slightly increased noise level could be seen for odd harmonics of the power line frequency of 50 Hz (150 Hz, 250 Hz, 350 Hz, et cetera). The noise floor for other frequencies was not critically affected (Figure 6).

### Discussion (Experiment 2)

Measurements of noise artifacts in MEG resulted in very sharp well-defined peaks at multiples of the operation frequency of the touchpad (600 Hz). Varying the pressure on the touchpad with a frequency of 1 Hz also caused an increase in noise activity in this frequency. Furthermore, some increase of noise power was observed at multiples of the power-line frequency of 50 Hz.

The artifacts at the operating frequency of this apparatus at 600 Hz and its harmonics at 1,200 Hz, 1,800 Hz, 2,400 Hz, etc., appear at frequency ranges that are usually not considered in neuroimaging experiments. In most neuromagnetic studies, the frequency of the brain responses of interest is below 150 Hz and thus the apparatus does not introduce major artifacts. Yet, strict care needs to be taken that these frequencies do not interact with auxiliary functions of the MEG system like the oscillatory signals used for continuous head localization. Artifacts due to local changes of pressure on the resistive touchpad and thus changes in the total touchpad current are less critical under normal operation because the total current is unchanged when moving the scanning probe across the touchpad with a defined force. In addition, artifacts due to the current flow in the touchpad can effectively be suppressed using 3rd order gradiometers. Furthermore, some increase of noise power was observed at multiples of the power-line frequency of 50 Hz. Since most MEG studies investigate frequencies below 150 Hz and since the neuromagnetic brain signals at the power line frequency and its harmonics are contaminated anyway, our device does not impose any additional or critical limitations for the study of brain magnetic activity related to active touch.

## Experiment 3: Neuromagnetic Activity Evoked by The Tactile Virtual Reality

To illustrate the potential and the feasibility of using the tactile virtual reality for neuroimaging studies we conducted an experiment on a single participant (male, 41 years old). The participant performed a distance discrimination task during which he was requested to indicate whether the distance between two ridges was smaller or larger than in the previous trial. This task required the integration of phasic sensory and persistent motor information and was therefore ideal to study active touch and compare it to a passive perception task. Here, we demonstrate the possibility of recording neuromagnetic signals without any interference from the equipment. We provide a glimpse of how the setup can contribute to the investigation of sensorimotor interaction by presenting somatosensory evoked neuromagnetic responses. Contrasting the magnetic activity obtained during active touch to the activity evoked by passive stimulation revealed differences in activation dynamics between both conditions. For reasons of article length, we do not emphasize the results of the behavioral aspect of the task and refrain from the description of the time-frequency results (Bauer et al., 2006).

### Method (Experiment 3)

#### Experimental Procedure

The distance discrimination task consisted of two conditions; both conditions were conducted in the same experimental session, first the active condition and then subsequently, the passive condition. In each trial of the active condition, the participant moved the scanning probe from the home position at the left margin of the touchpad to the right margin and back. During the movement, the participant encountered two virtual ridges. Because the participant scanned the virtual surface twice (i.e., left to right and back), this led to four tactile stimulations of the fingertip. Within a trial the distance between the two virtual ridges was constant, and the duration encountered by the participant in each direction of the movement depended completely on the participant’s movement kinematics. Whenever the scanning probe went over the virtual ridges, the vertical columns of the 4 × 4 piston matrix (i.e., along the long axis of the scanning probe, see Figure 1) were sequentially activated along columns such that it emulated the relative movement of the ridge across the fingertip. The sequential activation of the piston columns thus depended on the speed of the scanning movement. At the end of each trial, the participant indicated whether the distance between the two ridges was larger or smaller than that in the previous trial by button press; the participant did not receive any feedback on whether he was correct or not. The distances between the ridges varied from 30 mm to 50 mm in steps of 5 mm. To deter the participant from using specific veridical positions on the touchpad as a cue, we randomized the position of the ridges relative to the touchpad across trials. Each distance was presented at least 26 times resulting in a minimum of 520 stimuli per block. White noise was presented during the experiment to mask the sound generated during the activation of the piezo-elements. The activity of the piezo-elements was recorded and used as stimuli for the passive condition.

During the passive condition, the participant held the scanning probe stationary at the left margin of the touchpad (equivalent to the home position of the active condition). In the passive condition, stimuli were applied that were characterized by the same mean and standard deviation of the interstimulus intervals as in the active touch condition. Since the passive touch condition did not involve any hand movement, thereby no concept of distance estimation, the participant was asked to judge whether the interval (i.e., the difference in duration corresponding to the distance differences in the active task) between two tactile stimuli in the current trial was longer than that in the previous trial.

#### Neuromagnetic Recording

During the task, neuromagnetic data were simultaneously acquired with our 275 channel whole-head MEG system (CTF OMEGA, Port Coquitlam, Canada). Data were recorded at a sampling rate of 1,172 Hz, and an anti-aliasing filter set at 293 Hz. The onset of the piston movement served as a trigger for the time-locked analysis of the neuromagnetic data. Evoked magnetic fields elicited during the active exploration condition were compared to the passive stimulation. To demonstrate the recordability of evoked responses elicited by moving across the ridges, somatosensory evoked fields were computed by averaging stimulus-locked brain responses across trials. For each trial in the active condition, we obtained four evoked responses—two ridges (i.e., the distance boundaries) were encountered during both, the left to right movement, and the same two ridges during the returning direction. In the passive condition, the hand was kept stationary and evoked responses were obtained by applying tactile stimuli with interstimulus intervals whose mean and standard deviation were the same as in the active version. At the end of each trial in the passive condition, the participant had to indicate whether the “distance” in the current trial was shorter/longer than that in the previous trial. Because this condition is a temporal discrimination task, the decision of whether subsequent trials differ is based on the perceived duration estimates between the first and the second, and the third and fourth stimuli of each trial. In both, the active and passive conditions, the sound of the tactile stimulation was masked with white noise presented binaurally.

### Results (Experiment 3)

**Figure 7:**
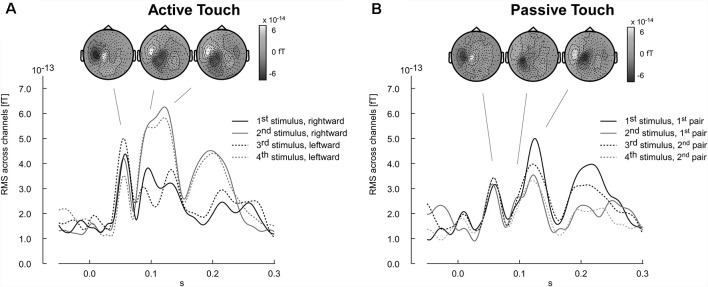
Time courses of neuromagnetic stimulation following the onset of the tactile stimulation (time point zero). Each wave shape represents the root mean square (RMS) of the neuromagnetic activity across channels. **(A)** Active touch condition: first and second stimuli refer to the ridges encounter during the rightward movement. The third and fourth stimuli refer to the leftward return movement. **(B)** Passive touch condition: the stimulus number refers to the sequence as it was delivered to the fingertip. Respective topographies presented as insert reference to the individual components averaged across all stimulus-evoked instances (stimulus 1–4). Positive magnetic fields appearing as white regions in the topographical map represent magnetic field lines leaving the head (outgoing). In contrast, negative magnetic activity is depicted in black and indicates magnetic field lines entering the head (ingoing).

The results of the pilot experiment show that the evoked magnetic field can be recorded using the equipment designed as tactile virtual reality. Stimulation artifacts are negligible and did not corrupt major components of the somatosensory evoked fields, in particular with latencies larger than 50 ms. Furthermore, the participant’s pilot data revealed a difference in the evoked response between active and passive touch. Amplitudes of the M60 component were larger in the active touch condition than in the passive condition (Figure 7). Furthermore, the evoked response pattern for latencies ranging between 80 and 150 ms appeared to be more complex in the active than in the passive condition (Figure 7). In the active condition, a systematic variation of the wave shapes across the four edge-evoked responses is present (Figure 7A).

### Discussion (Experiment 3)

The topography of the M60, M100 and M120 components for all four stimulus conditions shows the typical pattern of ingoing and outgoing magnetic fields that are expected for right finger stimulation activating primary (M60) and secondary (M100, M120) somatosensory cortex (Figure 7). For the interest of this method article we only provide the MEG data of one pilot participant. The example shows that there are no major artifacts corrupting the MEG recording and that the setup enables ecologically relevant scenarios.

## Experiment 4: Steady-State Responses in Active Touch

Scanning a structured surface by exploratory hand and finger movements generate a steady somatosensory input. While steady-state responses to somatosensory stimuli being delivered at a fixed frequency have been intensively studied in electroencephalography (EEG) and MEG (Narici et al., 1987; Snyder, 1992; Vakorin et al., 2010), the recording of movement-induced tactile steady-state responses suffers from variable stimulation frequencies. Experiment 4 demonstrates that despite these shortcomings steady-state responses can be successfully retrieved from continuously recorded MEG brain signals.

### Method (Experiment 4)

#### Experimental Setup

Movement-induced steady-state responses were studied in a single participant (male, 41 years). In the task, regularly spaced virtual gratings were used as stimuli. The ridges of the virtual grating had a width of 2.5 mm wide and a spacing of 10 mm. Whenever the scanning probe reached a position on the touchpad which was associated with virtual ridges the corresponding columns of pistons of the piezo-stimulator inbuilt in the scanning probe were activated. Moving over the virtual ridge neighboring columns of the piston matrix of the scanning probe were activated sequentially, mimicking the moving of the ridge across the fingertip during the hand movement. On the touchpad surface, there were 30 ridges in total. The participant was instructed to move the scanning probe across the touchpad from left to right and back again for 20 s per trial with his right hand. The participant was informed to stay within the central 300 mm of the 400 × 325 mm touchpad in order not to interrupt the sensory input when moving to the outer borders of the pad. Overall there were three training trials and 21 trials during which neuromagnetic responses were recorded. During training trials, the participant was familiarized with the task. He was instructed to find the speed at which the gratings could be perceived best and to maintain the same speed for the subsequent trials during which the magnetic oscillatory steady-state responses were recorded. Whenever the pistons were activated, a trigger was generated to monitor the stimulation of the participant’s index finger.

#### Neuromagnetic Recording

Neuromagnetic steady-state responses were recorded using a 275 channel MEG system (CTF OMEGA, Port Coquitlam, Canada) with a sampling rate of 11.719 kHz, and an anti-aliasing filter set at 2.93 kHz in order record short-latency activity. The onset of the piston movement served as a trigger for synchronizing the recording to the stimulation. In addition to brain signals, the piezo control signal determining the protrusion of pistons and the trigger signal indicating the onset of the tactile stimuli were recorded for 7 min.

#### Data Analysis

**Figure 8:**
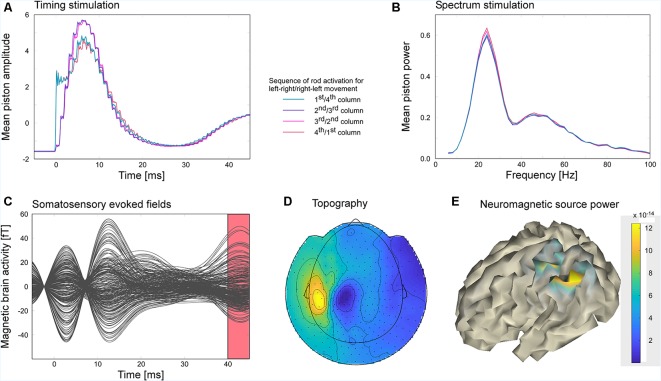
The participant explored the virtual gratings of a touchpad surface by moving the scanning probe unit horizontally across the touchpad with his right hand. Electrically controlled piezo pistons arranged in a 4 × 4 matrix stimulated the tip of the right index finger depending on the position of the scanning probe on the touchpad. Moving across a virtual ridge of the grating activated the vertical columns of the piston matrix sequentially in order to mimic the sliding of the finger over the ridge. **(A)** Average piston control signal. Depending on movement direction the left-most column of the piston matrix was either activated first or last. Vice versa the right-most column was either activated last or first. Middle columns were always activated second or third. **(B)** Spectra of the piston control signals indicate that the stimulation frequency varied considerably across time. **(C)** The steady-state activity was segmented in 7,138 stimulus-locked snippets of 50 ms duration. Averaged snippets resulted in an evoked brain response representing the steady-state activity. **(D)** Dipolar topographical distribution of the evoked brain activity corresponding to the time window of the evoked response shown as red interval in panel **(C)**. **(E)** Evoked brain activities were localized on a cortical surface using a minimum norm approach. Increased activity was found in the postcentral and posterior parietal cortex.

Since the movement-induced stimulation frequency varied in a range extending from 16 to 32 Hz (see Figure 8B), the steady-state response could not be extracted by filtering the MEG-data in the frequency of the stimulation but was extracted as an evoked response following the onset of the tactile stimuli. Using the MatLab toolbox fieldtrip (Oostenveld et al., 2011), neuromagnetic data were filtered with an 80 Hz lowpass filter and cut into 7,138 segments of 50 ms duration with a prestimulus baseline of 5 ms and a poststimulus period of 45 ms. The mean amplitude of the poststimulus interval ranging from 40 to 45 ms was projected on the cortical sheet of the individual subject, by means of a minimum norm approach. For regularization, the covariance matrix of the baseline interval was used (regularization parameter: λ = 3). The cortical sheet used for source localization was derived from the participant’s MR (1 mm, isotropic T1-weighted whole-head structural image, 3D-MPRAGE, TR 2.3 s, TE 3.03 ms, FA 8°) by means of the toolboxes Freesurfer (Dale et al., 1999; Fischl et al., 1999) and SUMA (Saad and Reynolds, 2012; Li Hegner et al., 2018). In order to investigate how stimuli were delivered during the movement of the scanning probe, the piezo control signal for each piston matrix column was averaged across trials. Furthermore, the piezo control signal was cut into segments of 1 s duration and subjected to a frequency analysis estimating the power spectrum in a frequency range of 5–100 Hz in steps of 5 Hz. The frequency analysis used a set of Slepian sequences as tapers with a smoothing of ±2.5 Hz (Oostenveld et al., 2011).

### Results (Experiment 4)

The power spectrum of the piezo control signal (see Figure 8B) revealed that the speed of the scanning probe movement varied considerably leading to stimulation frequencies in the range between 10 and 38 Hz with a peak maximum at 24 Hz. The peak of the average piezo control signals was reached at about 6.4 ms. On average the different columns of the piston matrix of the piezo stimulator were similarly activated except that the activation of the first and last column revealed a faster rise time (see Figure 8A). Movement-induced somatosensory evoked responses revealed two peaks at 2.8 and 12.5 ms and as well at 42.7 ms (Figure 8C). While the early peaks most likely reflected the stimulation artifact, the later component exhibited activation in the left centro-parietal hemisphere (Figure 8D), contralateral to the stimulation side. The blue and yellow patches in Figure 8D represent the in- and outgoing magnetic fields of the underlying dipolar source. Minimum norm source analysis revealed an activated cortical area that extends from postcentral to the posterior parietal cortex (Figure 8E).

### Discussion (Experiment 4)

Experiment 4 revealed that movement-induced somatosensory steady-state response can be successfully recorded using the here presented tactile virtual reality. Although peaks around the stimulation onset most likely reflect residual stimulation artifacts, the evoked response was clear and yielded a dipolar pattern which is typical for the activation of primary somatosensory cortex and higher associative sensory brain regions. Due to the movement-dependent stimulation the frequency at which stimuli were presented varied between 10 and 38 Hz. Due to the large variability of the stimulation frequency, band-pass filtering the data at the stimulation frequency did not result in a clear topography of the steady-state response. In contrast, analyzing the steady-state activity as the evoked activity phase-locked to the 7,138 stimulus presentations yielded a well-defined response pattern. The source of the steady-state response included the primary somatosensory cortex in post-central sulcus and extended to the posterior parietal cortex. The involvement of the posterior parietal cortex might reflect the integration of sensory and motor information that is needed to infer the features of the surface texture (Mohan et al., 2019).

## General Discussion: Conclusion and Perspectives

A bias-free and efficient estimation of vibrotactile threshold becomes especially important in clinical applications such as the diagnosis of patients suffering from central or peripheral neuropathy. For example, diabetes is known to affect peripheral nerve condition (Hyllienmark et al., 1995), and thus might affect the tactile sensory system (Travieso and Lederman, 2007).

Combining a tactile piezoelectric stimulator with a resistive touchpad, we assembled a virtual tactile reality that enables the investigation of active touch. The objective of the current study is to provide a proof of concept for the applicability and effectiveness of the novel apparatus. We demonstrated successfully the functionality of our device in four psychophysical and neuroimaging experiments.

In a first experiment, we estimated vibrotactile detection thresholds while participants actively scanned a virtual textured surface using their index finger. Although thresholds differed from values obtained with other methods, thresholds were consistent for both, the “active” and “passive” threshold estimation procedure. Whether the texture used in the current task was too simple to obtain better sensitivity in the active condition as compared to the passive condition needs to be clarified in future studies. Given the little overlap between the threshold values of Experiment 1 and the published norm values, we are currently unable to link our threshold values to the receptor types described in the literature. In order to further develop the device, future studies are suggested to explore the setup systematically by varying the spatial frequency of the virtual textures and by stimulating the fingertip using a high-resolution piston matrix. In addition, upcoming experiments should investigate how different receptor types can be selectively stimulated using different stimulation parameters. For example, applying a random dot pattern as a stimulation surface and stimulating a larger skin area by using the complete stimulation matrix of all 16 pistons might selectively activate Pacinian corpuscles. Slowly adapting Merkel cells might be best stimulated by broader strips of protruded stimulus pistons inducing lower stimulation frequency than more narrow ridges (Bolanowski et al., 1988).

The participants’ performance and the integrity of the vibrotactile stimulus processing pathway is usually characterized by the thresholds of various stimulus parameters, such as stimulus intensity, frequency, roughness, etc. Current standard methods to test somatosensory detection and discrimination thresholds involve von-Frey hair probes (Braun et al., 2005), custom-built non-standardized procedures (Phillips et al., 1990), raised alphabets (Vega-Bermudez et al., 1991), tactile plates of varying spatial frequencies (Cascio and Sathian, 2001; Gamzu and Ahissar, 2001), just to name a few. The lack of any standard makes it difficult to compare and interpret the results across different studies. In addition, existing equipment for sensory testing in clinical applications often does not provide highly automatized and standardized procedures of stimulus presentation, which makes it difficult to obtain unbiased results.

The presented device could be considered an initial attempt towards creating an automated and bias-free approach to efficiently examine somatosensory performance in clinical settings. The objective to develop sensitive equipment is critical for the study of somatosensation in patients, particularly those suffering from peripheral neuropathy in diabetes (Christensen, 1969), hand-arm vibration syndrome (Bovenzi et al., 1999; Lindsell and Griffin, 1999), age- or disease-related loss of tactile sensitivity (Liu et al., 2002; Duke et al., 2007), carpal tunnel syndrome (Gandhi et al., 2012), reduced attentional span or in patients who have multiple impairments that might affect their performance in the lengthy threshold estimation procedures. The estimation of patients’ thresholds is usually implemented by asking the patients to rate the intensity or discriminability of the stimuli directly after each application. While such procedures are time-consuming, stimulation with our new method could serve as an efficient tool for the estimation of thresholds in a clinical environment. Finding the point on the virtual texture at which the protrusion of pistons is just noticeable might be a more effective way to study sensory performance in patients. Since one most important function of the somatosensory system is the exploration of textures by active touch, our virtual tactile reality might provide ecologically more relevant results than current standard procedures. Evidently, prior to any clinical use, the validity of the new procedure and its comparability and consistency with established procedures need to be verified.

Next, to demonstrate the usability of the setup as a stimulus delivery system during neuroimaging study, we investigated the noise introduced by our device to MEG recordings. Finally, in order to demonstrate the quality of the recorded data and the potential for the investigation of active touch, we conducted MEG measurements while the participant was performing the distance estimation task and the steady-state response task. The MEG results provide convincing evidence that our novel tactile virtual reality setup does not corrupt neuroimaging signals by noise. Results obtained for the acquisition of neuromagnetic activity revealed brain activities contralateral to the stimulated hand, both, for event-related and steady-state conditions. Artifacts appeared specifically at the frequency of operation of the touchpad. Since these frequencies are known, they can be eliminated during data analysis. Using an operating frequency for the touchpad of 600 Hz shifts the artifacts into a frequency range which is clearly defined and far from most of the oscillatory brain activities under investigation. Despite the sources of noise that are inherent to the virtual reality setup, the presented experiments provide a first convincing proof of principle that the equipment has the potential to get insight into how the sensory and motor systems interact in active touch.

Although in the here presented experiments we have used the scanning probe only in left to right and right to left direction, ridges with any arbitrary direction can also be presented in order to explore a multitude of textures. In general, a wide range of virtual surfaces with different degrees of complexity can be programmed. Thereby, the extension of the surface texture is defined by the size of the touchpad. Since the number of stimulation pistons can be potentially increased, the spatial resolution of the stimulation matrix can be improved further. Moreover, various layouts of piston arrangements can be configured that might allow for more refined stimulations. Currently, stimulation pistons are retracted by gravity. Fixating the pistons in their retainers by elastic filaments would even enable the exploration of textures placed in any direction in space. Given these possible developments, the here presented equipment should be only regarded as a first prototype.

Concluding, the capability to quickly determine somatosensory thresholds the new device opens a wide perspective to study both, multisensory integration, and sensorimotor gating. It will allow detailed investigations of the interaction between hand movements and somatosensation in active touch. More importantly, its compatibility with neuroimaging methods such as MEG will furthermore enable the extension of somatosensory research towards the investigation of neural correlates of active scanning of surface textures.

## Data Availability Statement

The datasets generated for this study are available on request to the corresponding author.

## Ethics Statement

The study was in full accordance with the Frontiers authors guidelines. The study was evaluated by the Ethics Board of the Medical Faculty of the University of Tübingen.

## Author Contributions

AB has designed the somatosensory threshold paradigm, has written parts of the manuscript and handled the revision of the manuscript. DK has analyzed the data and has written the manuscript. AP has collected the data and provided the initial drafts of the manuscript. YL has contributed to the data analysis. MZ has provided the project idea and has edited the manuscript. CS has provided critical comments and valuable contributions to the manuscript. CB is responsible for the technical realization of the project. He has written parts of the manuscript.

## Conflict of Interest

The authors declare that the research was conducted in the absence of any commercial or financial relationships that could be construed as a potential conflict of interest.
